# Supplementary material for: Identification of Tumor Suppressive Genes Regulated by miR-31-5p and miR-31-3p in Head and Neck Squamous Cell Carcinoma
Source: Int J Mol Sci. 2021 Jun 8;22(12):6199. doi: 10.3390/ijms22126199 (PMC8227492; doi:10.3390/ijms22126199)
Supplement: Supplementary file 1 [file ijms-22-06199-s001.zip › ijms-1216243-supplementary figures.pptx]

## Slide 1
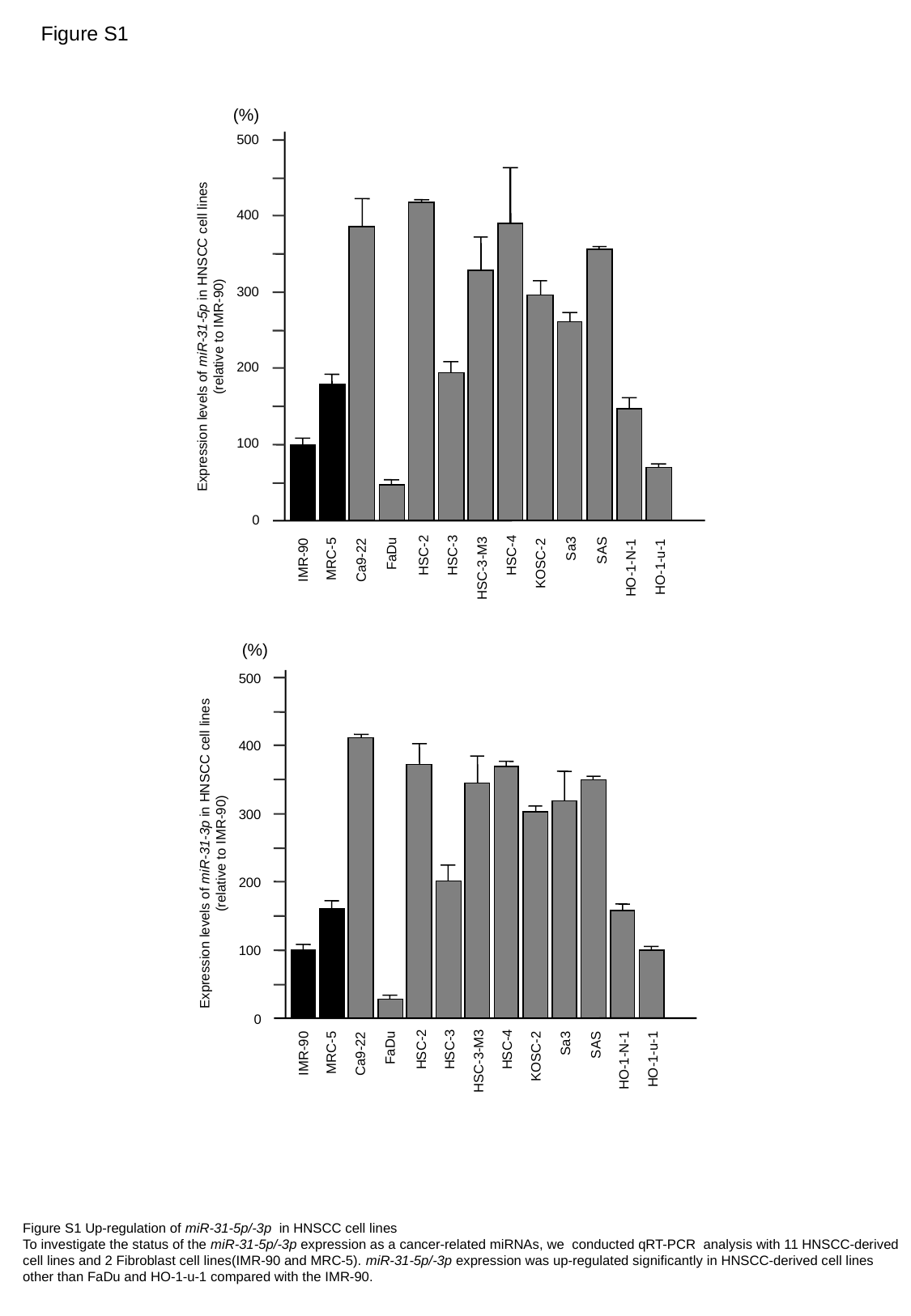

Figure S1
(%)
500
400
300
Expression levels of miR-31-5p in HNSCC cell lines
(relative to IMR-90)
200
100
0
Sa3
SAS
FaDu
HSC-3
HSC-2
HSC-4
MRC-5
IMR-90
Ca9-22
KOSC-2
HO-1-u-1
HO-1-N-1
HSC-3-M3
(%)
500
400
300
Expression levels of miR-31-3p in HNSCC cell lines
(relative to IMR-90)
200
100
0
Sa3
SAS
FaDu
HSC-3
HSC-2
HSC-4
MRC-5
IMR-90
Ca9-22
KOSC-2
HO-1-u-1
HO-1-N-1
HSC-3-M3
Figure S1 Up-regulation of miR-31-5p/-3p in HNSCC cell lines
To investigate the status of the miR-31-5p/-3p expression as a cancer-related miRNAs, we conducted qRT-PCR analysis with 11 HNSCC-derived cell lines and 2 Fibroblast cell lines(IMR-90 and MRC-5). miR-31-5p/-3p expression was up-regulated significantly in HNSCC-derived cell lines other than FaDu and HO-1-u-1 compared with the IMR-90.

## Slide 2
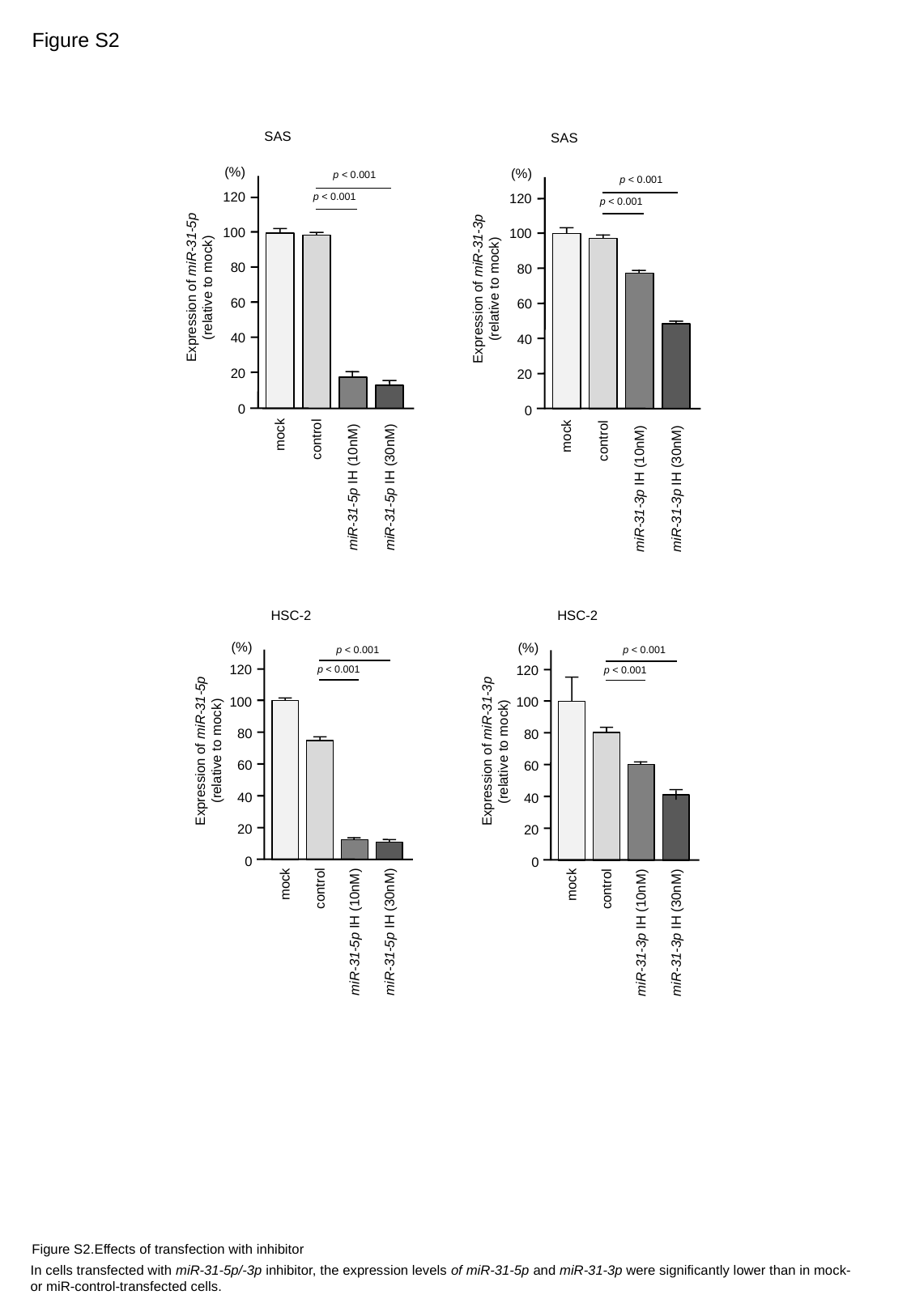

Figure S2
SAS
p < 0.001
(%)
p < 0.001
120
100
80
Expression of miR-31-5p
(relative to mock)
60
40
20
0
mock
control
miR-31-5p IH (10nM)
miR-31-5p IH (30nM)
SAS
(%)
p < 0.001
p < 0.001
120
100
80
Expression of miR-31-3p
(relative to mock)
60
40
20
0
mock
control
miR-31-3p IH (10nM)
miR-31-3p IH (30nM)
HSC-2
p < 0.001
(%)
p < 0.001
120
100
80
Expression of miR-31-5p
(relative to mock)
60
40
20
0
mock
control
miR-31-5p IH (10nM)
miR-31-5p IH (30nM)
HSC-2
p < 0.001
(%)
p < 0.001
120
100
80
Expression of miR-31-3p
(relative to mock)
60
40
20
0
mock
control
miR-31-3p IH (10nM)
miR-31-3p IH (30nM)
Figure S2.Effects of transfection with inhibitor
In cells transfected with miR-31-5p/-3p inhibitor, the expression levels of miR-31-5p and miR-31-3p were significantly lower than in mock- or miR-control-transfected cells.

## Slide 3
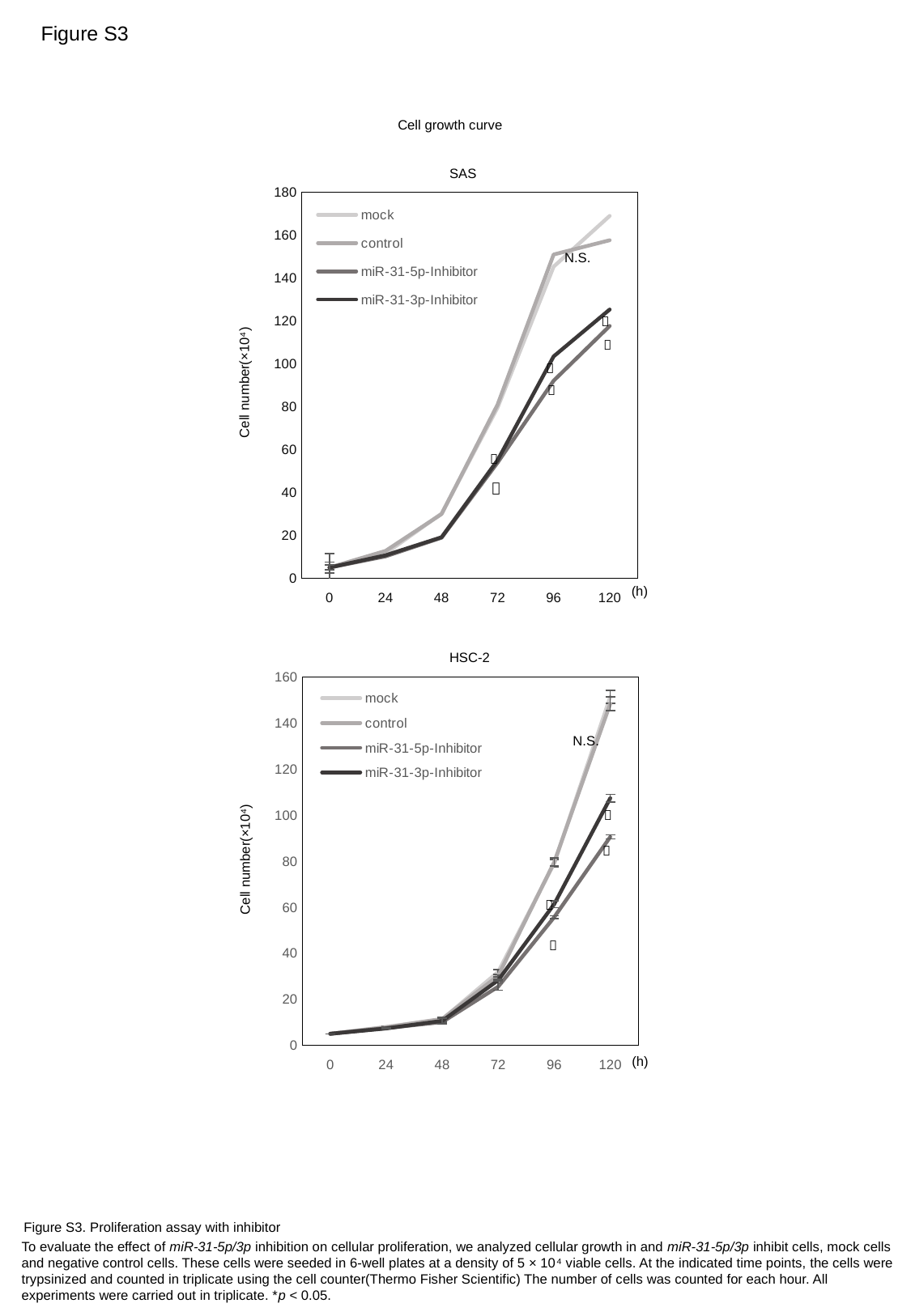

Figure S3
Cell growth curve
### Chart
| Category | mock | control | miR-31-5p-Inhibitor | miR-31-3p-Inhibitor |
|---|---|---|---|---|
| 0 | 5.0 | 5.0 | 5.0 | 5.0 |
| 24 | 11.526666666666666 | 12.75 | 10.226666666666667 | 10.7 |
| 48 | 30.166666666666668 | 29.933333333333334 | 18.933333333333334 | 19.133333333333333 |
| 72 | 79.63333333333333 | 81.0 | 53.93333333333334 | 55.03333333333333 |
| 96 | 145.33333333333334 | 151.0 | 92.13333333333333 | 103.46666666666665 |
| 120 | 169.0 | 157.66666666666666 | 117.66666666666667 | 125.33333333333333 |＊
＊
＊
Cell number(×10⁴)
＊
N.S.
(h)
SAS
＊
HSC-2
### Chart
| Category | mock | control | miR-31-5p-Inhibitor | miR-31-3p-Inhibitor |
|---|---|---|---|---|
| 0 | 5.0 | 5.0 | 5.0 | 5.0 |
| 24 | 8.006666666666666 | 7.81 | 7.466666666666666 | 7.32 |
| 48 | 11.333333333333334 | 11.45 | 10.083333333333334 | 10.623333333333333 |
| 72 | 31.900000000000002 | 30.2 | 25.46666666666667 | 28.266666666666666 |
| 96 | 79.06666666666666 | 79.6 | 55.666666666666664 | 61.333333333333336 |
| 120 | 151.33333333333334 | 148.33333333333334 | 90.53333333333335 | 107.33333333333333 |N.S.
＊
＊
Cell number(×10⁴)
＊
＊
(h)
Figure S3. Proliferation assay with inhibitor
To evaluate the effect of miR-31-5p/3p inhibition on cellular proliferation, we analyzed cellular growth in and miR-31-5p/3p inhibit cells, mock cells and negative control cells. These cells were seeded in 6-well plates at a density of 5 × 10⁴ viable cells. At the indicated time points, the cells were trypsinized and counted in triplicate using the cell counter(Thermo Fisher Scientific) The number of cells was counted for each hour. All experiments were carried out in triplicate. *p < 0.05.

## Slide 4
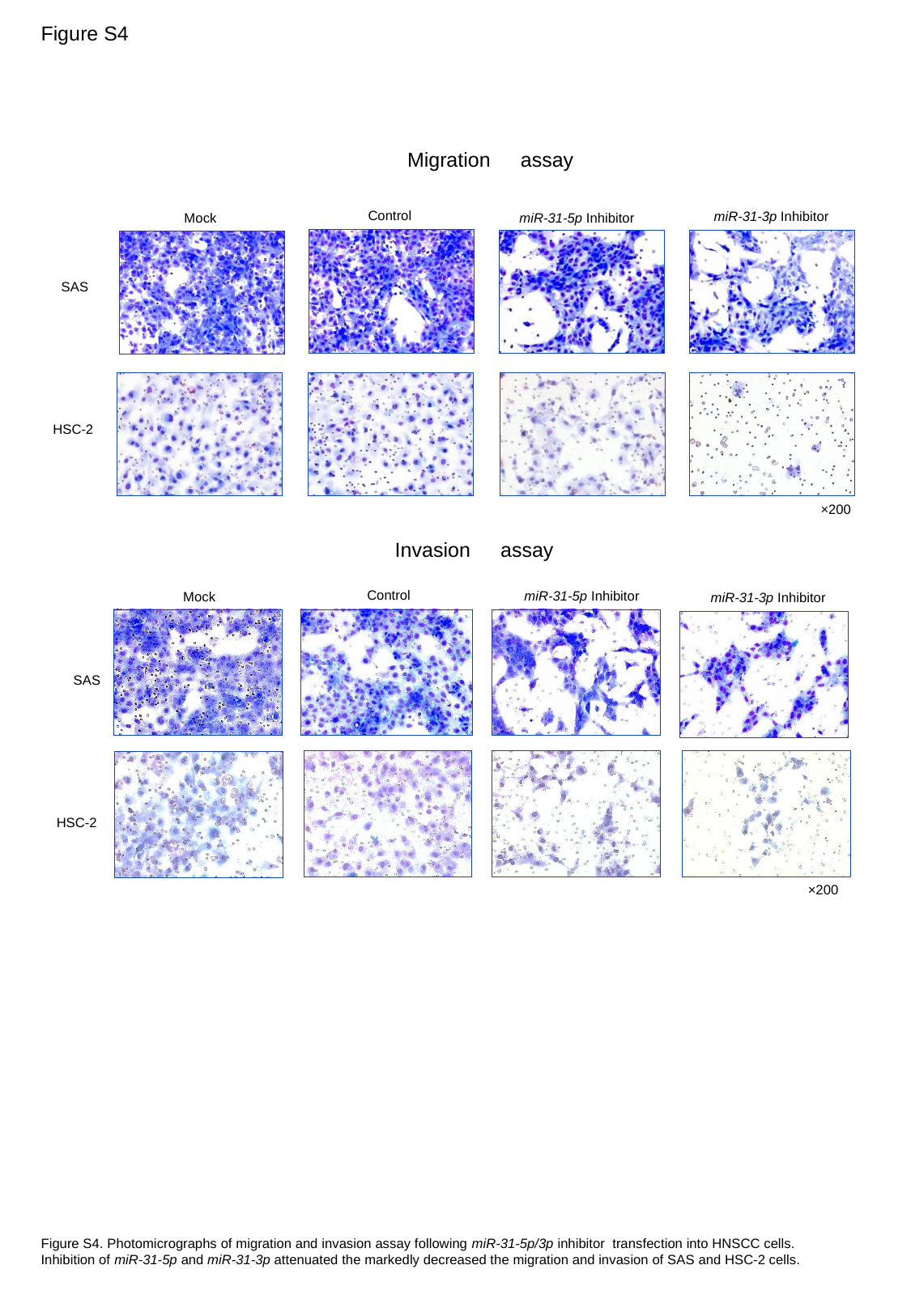

Figure S4
Migration　assay
Control
miR-31-3p Inhibitor
Mock
miR-31-5p Inhibitor
SAS
HSC-2
×200
Invasion　assay
Control
miR-31-5p Inhibitor
Mock
miR-31-3p Inhibitor
SAS
HSC-2
×200
 Figure S4. Photomicrographs of migration and invasion assay following miR-31-5p/3p inhibitor transfection into HNSCC cells.
 Inhibition of miR-31-5p and miR-31-3p attenuated the markedly decreased the migration and invasion of SAS and HSC-2 cells.
